# Adoptive Immunotherapy of Cytokine-Induced Killer Cell Therapy in the Treatment of Non-Small Cell Lung Cancer

**DOI:** 10.1371/journal.pone.0112662

**Published:** 2014-11-20

**Authors:** Min Wang, Jun-Xia Cao, Jian-Hong Pan, Yi-Shan Liu, Bei-Lei Xu, Duo Li, Xiao-Yan Zhang, Jun-Li Li, Jin-Long Liu, Hai-Bo Wang, Zheng-Xu Wang

**Affiliations:** 1 Biotherapy Center, General Hospital of Beijing Military Command, Beijing, China; 2 Department of Biostatistics, Peking University Clinical Research Institute, Peking University Health Science Center, Beijing, China; University of Pittsburgh, United States of America

## Abstract

**Aim:**

The aim of this study was to systemically evaluate the therapeutic efficacy of cytokine-induced killer (CIK) cells for the treatment of non-small cell lung cancer.

**Materials and Methods:**

A computerized search of randomized controlled trials for CIK cell-based therapy was performed. The overall survival, clinical response rate, immunological assessment and side effects were evaluated.

**Results:**

Overall, 17 randomized controlled trials of non-small cell lung cancer (NSCLC) with a total of 1172 patients were included in the present analysis. Our study showed that the CIK cell therapy significantly improved the objective response rate and overall survival compared to the non-CIK cell-treated group. After CIK combined therapy, we observed substantially increased percentages of CD3^+^, CD4^+^, CD4^+^CD8^+^, CD3^+^CD56^+^ and NK cells, whereas significant decreases were noted in the percentage of CD8^+^ and regulatory T cell (Treg) subgroups. A significant increase in Ag-NORs was observed in the CIK-treated patient group (*p* = 0.00001), whereas carcinoembryonic antigen (CEA) was more likely to be reduced to a normal level after CIK treatment (*p* = 0.0008). Of the possible major side effects, only the incidence of fever in the CIK group was significantly higher compared to the group that received chemotherapy alone.

**Conclusion:**

The CIK cell combined therapy demonstrated significant superiority in the overall survival, clinical response rate, and T lymphocytes responses and did not present any evidence of major adverse events in patients with NSCLC.

## Introduction

Lung cancer is the leading cause of cancer-related mortality worldwide [1]. According to the 2012 Chinese cancer registration annual report, more than 3 million new cases of lung cancer will be diagnosed every year, and the approximately 2.7 million deaths from lung cancer will account for 13% of allmortalities. There is no doubt that the incidence and mortality of lung cancer are far too prevalent [2]. In patients with advanced lung disease, 1-year survival rates are typically 35%, and 2-year survival rates were shown to approach 15%-20% in recent studies [3]. At best, the 5-year overall survival rate of localized cancer is 15.9%, and only half of extended-stage patients have a 3.7% chance of surviving 5 years [4]. Most NSCLC patients have locally advanced or metastatic cancer at stage IIIB-IV at the time of diagnosis, leaving only palliative therapeutic options. Based on the existing clinical data, chemotherapy appears to have limited benefits and disappointed prognoses [5].

The novel approach of adoptive cell immunotherapy relies on an ex vivo expansion of the autologous tumor-specific effector cells before their reinfusion into the host [6]. Since the development of this immunotherapy, a number of immunological effector cells have been employed to treat cancer and eliminate residual tumor cells after surgery, such as CIK cells, lymphokine-activated killer cells (LAKs), tumor-infiltrating lymphocytes (TILs), natural killer cells (NKs), and cytotoxic T lymphocyte cells (CTLs) [7], [8]. Among them, LAKs, which are a mixture of lymphokine-activated CD3^+^ T lymphocytes and CD3^−^CD56^+^CD16^+^ NK cells, were cultured with recombinant interleukin-2 (rIL-2) for 3 days, and CTLs were isolated from a patient's own tissues, including peripheral blood mononuclear cells (PBMCs), TILs, draining lymph nodes, or PBMCs after vaccination with irradiated autologous tumor cells (ATCs) [7], [8]. After adoptive cell immunotherapy made great strides due to the efforts of several generations of researchers, CIK cells were found to possess greater proliferative and cytolytic capacities than NK or LAK cells. CIK cells are MHC-unrestricted cytotoxic lymphocytes that can be generated in vitro from PBMCs and cultured with the addition of IFN-γ, IL-2 and CD3 monoclonal antibody (CD3mAb). Anti-tumor cytotoxic activity is represented by surface markers for both T cells (TCR-α/β, CD3) and NKT cells (CD3^+^CD56^+^) [9].

The first clinical trial using CIK cell therapy for cancer patients was reported in 1999 [10]. Soon afterward, a growing number of clinical trials have suggested that CIK therapy yields highly compelling objective clinical responses in several solid carcinomas compared to other immunological effectors. A pooled analysis of 792 patients with solid carcinomas indicated that treatment with CIK cells is associated with a significant prolonging of the mean survival time and disease control rate [11]. Recently, both chinese clinical trials with 563 patients and international registered clinical trials with 426 cases of CIK cell therapy provided evidence for a broad clinical application based on a positive evaluation of the immunological and clinical responses [12], [13]. Some systematic reviews have analyzed CIK cell therapy and shown it to be safe and efficient to treat renal cell carcinoma, hepatocellular carcinoma, and colon cancer [14]–[16]. Furthermore, CIK cell therapy has been perceived to have significant survival benefits in a few NSCLC clinical trials [17]–[22]. These studies showed that the immunotherapy of cancers with CIK cells may improve immunological and clinical responses, promote the quality of life (QoL) of cancer patients, and extend their life spans under certain conditions. However, there is no systematic review to assess the therapeutic efficacy of CIK cell therapies combined with chemotherapy in NSCLC; therefore, we performed a systematic meta-analysis of CIK cell therapy with randomized controlled trials on NSCLC. Our large-scale CIK cell immunotherapy clinical trials systematically analyzed the clinical efficiency and safety considering the overall survival, clinical response, immunological assessments and side effects.

## Methods

### Study design, search strategy and eligibility criteria

The relevant studies were identified by searching PubMed, the Cochrane Center Register of Controlled Trials, Science Direct, Embase, and China National Knowledge Infrastructure for randomized controlled trials (RCT) in the most recent decades. The search strategy included the keywords ‘non-small-cell lung cancer,’ ‘adoptive immunotherapy,’ and ‘cytokine induced killer cells’ adoptive immunotherapy arms with no adjuvant treatment in NSCLC patients except those who had undergone the same chemotherapy compared with control arms. In addition, we manually searched a website of clinical trials for ongoing trials. We searched keywords ‘non-small-cell lung cancer’ and ‘cytokine induced killer cells’ on the website http://www.clinicaltrials.gov/. The registered clinical trials with publication citations are displayed at the bottom of the Full Text View tab of a study record, under the More Information heading. Reference lists of previously published trials and relevant review articles were examined for other eligible trials. No language restriction was applied. Review papers and postgraduate theses were also examined for published results. Furthermore, we performed manual searches in reference lists and conference proceedings of the American Society of Clinical Oncology (ASCO) annual meetings and the European Cancer Conference (ECCO). We excluded abstracts that were never subsequently published as full papers and studies on animals and cell lines.

### Data selection criteria

Data extraction was independently conducted by two reviewers (Min Wang and Jun-Xia Cao) using a standardized approach. Disagreement was adjudicated by a third reviewer (Zheng-Xu Wang) after referring back to the original publications. The selection criteria were as follows: (1) English language studies on human clinical trials with patients at all stages of NSCLC were included; (2) RCT with CIK cell-based immunotherapy combined with chemotherapy versus chemotherapy alone for the treatment of NSCLC were included; (3) all trials approved by the local ethical committee and in which all patients signed a study-specific consent form prior to study entry were included; (4) case studies, review articles, and studies involving fewer than 10 patients were excluded; (5) uncontrolled metabolic disease, inadequate hepatic function, renal dysfunction, neurological disorders and other infectious diseases were excluded from the study; and (6) blood samples receiving any chemotherapy or radiotherapy within one month before treatment were excluded.

The overall quality of each included paper was evaluated by the Jadad scale [23]. A few of the major criteria were employed as a grading scheme: (1) randomization; (2) allocation concealment; (3) blinding; (4) lost to follow up; (5) ITT (intention to treat); and (6) baseline. We also used a funnel plot to evaluate the publication bias.

### Definition of outcome measures

The primary clinical endpoints in RCT for cancer therapies employed the measures of median survival time (MST) and progression-free survival (PFS). The time to progression (TTP) may not consider those patients who die from other causes but is often used as equivalent to PFS. The secondary endpoints were the clinical response rate, including the objective response rate (ORR) and disease control rate (DCR). The ORR was defined as the sum of the partial rates (PRs) and complete response rates (CRs), and the DCR was defined as the sum of the stable disease (SD), PR and CR, according to the World Health Organization criteria. The side effects and toxicity were graded according to the National Cancer Institute Common Toxicity Criteria. The data were either obtained directly from the articles or calculated using the graphed data in articles using Photoshop and a software graph digitizer scout.

### Statistical analysis

The analysis was performed using Review Manager Version 5.0 (Nordic Cochran Centre, Copenhagen, Denmark). Heterogeneity was assessed to determine which model should be used. To assess the statistical heterogeneity between the studies, the Cochran Q-test was performed using a predefined significance threshold of 0.1. The treatment effects are reflected by odds ratios (ORs), which were obtained using a method reported by Mantel and Haenszel. To evaluate whether the results of the studies were homogeneous, Cochran's Q test was performed. We also calculated the quantity I^2^, which describes the percentage of variation across studies that is due to heterogeneity rather than chance. The OR was obtained using a fixed-effect model with no statistically significant heterogeneity; otherwise, a random-effects model was employed. P-values <0.05 were considered statistically significant. All reported P-values were two-sided.

## Results

### Selection of the trials

**Figure 1:**
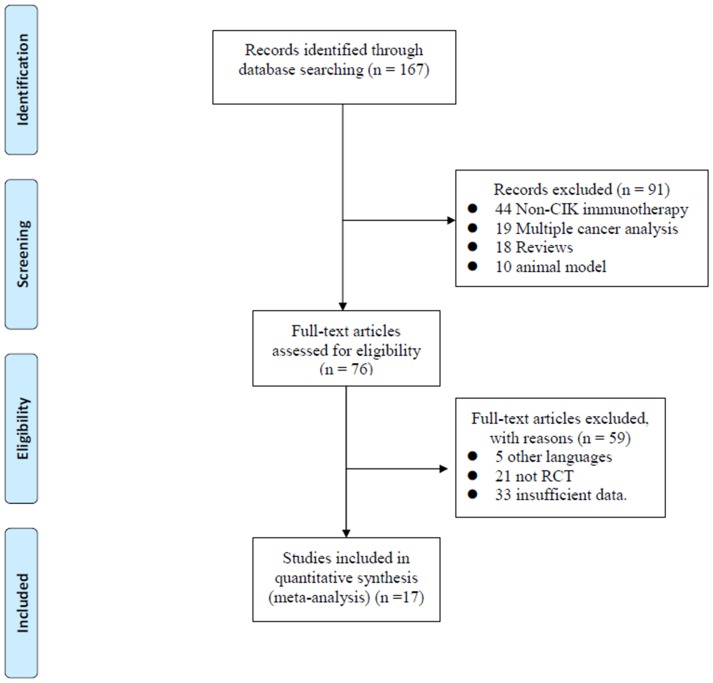
Flow diagram of the study selection process.

The data searches yielded 167 references, 91 of which were considered ineligible for different reasons (44 non-CIK immunotherapy, 19 multiple cancer analyses, 18 reviews, and 10 animal models). The remaining 76 articles were further evaluated, and 59 trials were excluded due to language, lack of an RCT, and insufficient data. The final 17 articles were included in the meta-analysis with RCTs of CIK cell-based therapy for the treatment of NSCLC (Figure 1, also see the checklist S1).

**Figure 2:**
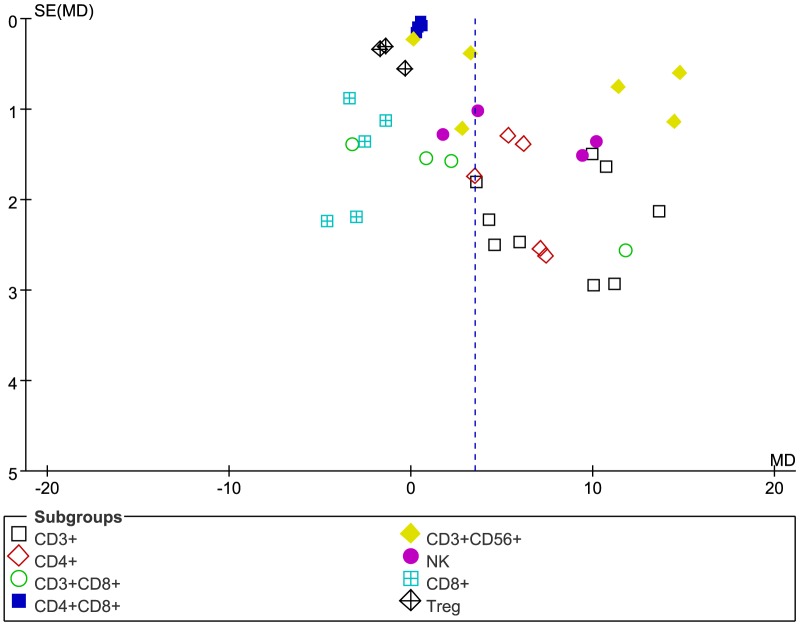
Funnel plot to evaluate the publication bias of T-cell subgroups. The analysis was performed using Review Manager Version 5.0.

The quality assessment of the 17 studies is summarized in Table 1. We also used a funnel plot to evaluate the publication bias. In our analysis, overall survival, clinical response rate, and side effects suffered low published bias. However, immunological assessment and T cell subgroups observed a high published bias (Figure 2), which demonstrated that the node of the vertical line does not meet the horizontal one at the midpoint by analysis with Review Manager Version 5.0.

**Table 1:** Jadad Scale for the 17 randomized controlled studies.

Included studies	Randomization	Allocation concealment	Blinding	Lost to follow up	ITT analysis	Baseline	Quality grading
Li 2009 [17]	Yes	Unclear	Unclear	No	Yes	Similar	B
Li 2012 [30]	Yes	Unclear	Unclear	No	Yes	Similar	B
Mo 2010 [18]	Yes	Unclear	Unclear	Yes	Unclear	Unclear	C
Peng 2012 [19]	Yes	Unclear	Unclear	No	Yes	Similar	B
Sheng 2011 [20]	Yes	Unclear	Unclear	No	Yes	Similar	B
Shi 2012 [21]	Yes	Unclear	Unclear	No	Yes	Similar	B
Wang 2013 [39]	Yes	Unclear	Unclear	Yes	Yes	Similar	B
Wu 2008 [6]	Yes	Unclear	Unclear	No	Yes	Similar	B
Xu 2010 [31]	Yes	Unclear	Unclear	No	Yes	Similar	B
Xu 2011 [32]	Yes	Unclear	Unclear	No	Yes	Similar	B
Yang 2013 [33]	Yes	Unclear	Unclear	No	Yes	Similar	B
You 2012 [34]	Yes	Unclear	Unclear	Yes	Yes	Unclear	B
Yuan 2011 [35]	Yes	Unclear	Unclear	Yes	Yes	Unclear	C
Zhang 2012 [36]	Yes	Unclear	Unclear	No	Yes	Similar	B
Zheng 2012 [38]	Yes	Unclear	Unclear	No	Yes	Similar	B
Zhong 2008 [37]	Yes	Unclear	Unclear	No	Yes	Similar	B
Zhong 2011 [22]	Yes	Unclear	No	No	Yes	Similar	B

ITT: intention-to-treat. A: adequate, with correct procedure; B: unclear, without a description of the methods; C: inadequate procedures, methods, or information.

Each criterion was graded as follows: Yes, adequate, with correct procedure; Unclear, without a description of the methods; No, inadequate procedures, methods, or information. Each involved study was graded as follows: A, studies with a low risk of bias and which were scored as grade of A for all items; B, studies with a moderate risk of bias, with one or more grades of B; and C, studies with a high risk of bias, with one or more grades of C.

### Characteristics of CIK cell-based therapy

The characteristics of the 17 trials are listed in Table 2. Our selected 17 trials with a total of 1172 NSCLC patients in stage I-IV were included in the present analysis, and 90% of them included metastatic or locally advanced NSCLC. The enrolled ages were between 28 and 82 years of age, with a median age greater than 50.

**Table 2:** Clinical information from the eligible trials in the meta-analysis.

Trials	Age	No. of pts	Operative method	Tumor Stage	CIK regimens	CIK culture	DC modification
Li 2009 [17]	40–80; (M61)	42;42	Chemo; Chemo+DC-CIK	I-IIIA	1.3×10^9^/course, 4 treatments at intervals of a month	X-Vivo 20, IL-1α, IL-2, IFN-γ, CD3	ATL (100 µg/ml)
Li 2012 [30]	UK	37; 37	Chemo; Chemo+ CIK	III-IV	13×10^9^/course, twice in a cycle, at least 3 cycles	X-Vivo 20,IL-1α, IL-2, IFN-γ, CD3	NO DC
Mo 2010 [18]	39–77; (M60)	20;21	Chemo; Chemo+ DC-CIK	IV	2–6×10^6^/course, 6 times every second day	RPMI1640, IL-1α, IL-2, IFN-γ, CD3mAb	NI-DC
Peng 2012 [19]	65–79; (M71)	23; 24	Chemo; Chemo+ DC-CIK	III-IV	1×10^10^–2×10^12^/course, 2–3 times a week, 7 days intervals for 4 cycles	CM, IL-1α, IL-2, IFN-γ, CD3	NI-DC
Sheng 2011 [20]	35–65; (M54)	33; 32	Chemo; Chemo+ DC-CIK	III-IV	5×10^9^/course, 4 treatments in a week for 2 weeks	RPMI1640, IL-1α, IL-2, IFN-γ, CD3	NI-DC
Shi 2012 [21]	UK	30; 30	Chemo; Chemo+ DC-CIK	III-IV	5 times every second day	RPMI1640, IL-1, IL-2, CD3	NI-DC
Wang 2013 [39]	UK	11; 11	CK; CK+ CIK	AS	2×10^10^/course, 2 courses in 2 months	UK	No DC
Wu 2008 [6]	38–78; (M60)	30; 29	Chemo; Chemo+ CIK	III-IV	1×10^9^/course, 5 times every second day	RPMI1640, IL-1α, IL-2, IFN-γ, CD3	No DC
Xu 2010 [31]	47–75; (M59.6)	40; 38	Chemo; Chemo+ DC-CIK	III-IV	1.6×10^9^/course, 2 times a week in next following 4–5 weeks	RPMI1640, IL-2, IFN-γ, CD3	NI-DC
Xu 2011 [32]	45–73; (M59)	40; 45	Chemo; Chemo+ DC-CIK	III	1.3×10^9^/course, 2 times a week in 5–6 weeks	RPMI1640, IL-2, IFN-γ, CD3	NI-DC
Yang 2012 [33]	28–82; (M63.5)	61; 61	Chemo; Chemo + DC-CIK	III-IV	1.2×10^9^/course, 30day intervals for 4 cycles	X-vivo 20, IFN-γ,IL-1α,IL-2,CD3McAb	ATL (100 µg/ml)
You 2012 [34]	M 52	50; 55	Chemo; Chemo+ DC-CIK	III-IV	5×10^9^/course, 4 times a cycle, 2–6 cycles	RPMI1640, IL-1α, IL-2, IFN-γ, CD3mAb	NI-DC
Yuan 2011 [35]	M 66	32; 32	Chemo; Chemo+ DC-CIK	AS	4 times a cycle	Unknown	NI-DC
Zhang 2012 [36]	35–72; (M57)	50; 50	Chemo; Chemo+ DC-CIK	III-IV	28day intervals for 2 cycles	GT-T551,IL-2, IFN-γ, CD3	NI-DC
Zheng 2012 [38]	M 59	36; 36	γK; γK +CIK	III	1×10^10^/course, 1 month intervals for 2 cycles	RPMI1640, IL-1α, IL-2,IFN-γ, CD3mAb	No DC
Zhong 2008 [37]	M 53.6	44; 22	Chemo; Chemo+ DC-CIK	IB	2 times in 4 days	UK	CEA PI-DC
Zhong 2011 [22]	40–65	14; 14	Chemo; Chemo+ DC-CIK	IIIB- IV	1–1.7×10^9^/course,30day intervals for 4 cycles	CM, IFN-γ, IL-2,CD3McAb	CEA, PI-DC (10 µg/ml)

M: median; UK: unknown; AS: advanced stage; Chemo: chemotherapy; CK: cyberknife; γK: γ-knife; NI-DC: non-impulsed DC; ATL: Autologous tumor lysate; PI-DC: peptide impulse DC; Pts: Patients. The selective data include the authors' names, year of publication, trial period, sample size per arm, regimen used, median or mean age of patients, cell preparation, CIK-based therapy treatment and information pertaining to the study design.

In all 17 trials, the control arm was chemotherapy or cyberknife alone, whereas the treatment arm was chemotherapy or cyberknife combined with CIK cell therapy. In each trial, all of the patients in the CIK group were treated identically to those in the chemotherapy group in terms of chemotherapy doses and cycles. In all 17 trials of the treatment arm, most of the patients were treated with CIK cells plus DC immunotherapy combined with chemotherapy, although patients in four of the trials were injected with CIK cells combined with chemotherapy [6], [30], [38], [39]. Most of the CIK groups used DCs without pulse, i.e., the DCs were only induced to become mature before co-culture with CIK cells. In 4 out of 17 studies, the DCs were injected while being pulsed with lung cancer antigens or tumor lysate [17], [22], [33], [37]. Some of the necessary cytokines were supplied in a culture of CIK, IL-2, IFN-γ, and CD3mAb in a variety of culture media. The patients received cell infusions of 1×10^9^ to 2×10^12^ cells per course, mostly at a 10^9^ order of magnitude. Most of the treatments with repeated CIK cell infusions were administered for at least 2 weeks, and some of them lasted over 1 month. The injected route for immunotherapy was mainly intravenous for CIK cells and via subcutaneous injection for DCs (File S1 and File S2).

### Survival

The patients in the CIK group had significantly prolonged MST compared with those in the non-CIK group (95%CI −7.45 to −0.66, *p* = 0.02) (Table 3). The results of the pooled analysis showed that the CIK arm significantly extended overall survival at the end of follow-up, compared with the non-CIK group (Table 4). Three subgroups of patients of the CIK cell-based therapy group at 1-year survival, 2-year survival, and 3-year survival presented significant survival benefits compared to the patients in the non-CIK group (OR 0.64, 95%CI 0.46–0.91, *p* = 0.01; OR 0.36, 95%CI 0.22–0.59, *p*<0.0001; OR 0.37, 95%CI 0.20–0.70, *p* = 0.002, respectively), which was consistent with the overall survival (OR 0.50, 95%CI 0.39–0.64, *p*<0.0001). Based on the results of our analysis, the short-term survival subgroup showed a significant difference at the 1-year and 2-year survivals. The 1-year survival for the 282 patients in the CIK group was 56%, whereas a slightly lower 1-year survival rate was found for the non-CIK group (45% of 278 patients). A significant difference was also demonstrated in the 2-year survival group, which was 43.22% for 236 patients in the CIK group and 27.47% of 233 patients without the CIK cell treatment. The long-term survival rates in the CIK group showed a slight decrease compared with the short-term survival rate; however, a significant difference in the long-term survival rates was found compared to the non-CIK group (*p* = 0.002).

**Table 3:** Comparison of MTTP, MST, and MPFS between the non-CIK and CIK groups.

Event	No. of Trials [Ref]	No. of pts Non-CIK CIK		Mean Difference	95% CI	P value	Heterogeneity (I^2^)
MTTP	4 [6], [21], [31], [36]	97	100	−1.59	−2.70 to −0.47	0.005	0%
MST	4 [6], [31], [32], [37]	154	134	−4.06	−7.45to −0.66	0.02	0%
MPFS	3 [21], [30], [37]	161	139	−4.69	−13.27to 3.89	0.28	56%

MTTP: median time to progression; MST: median survival time; MPFS: median progression-free survival; Pts: patients; 95%CI: 95% confidence interval; significant difference: P value <0.05.

**Table 4:** Comparison of OS, ORR and DCR between the non-CIK and CIK groups.

Event	No. of Trials [Ref]	No. pts of Non-CIK CIK		Odds Ratio (OR)	95% CI	P value	Heterogeneity (I^2^)
1 yr OS	8 [6], [18], [19], [22], [31], [32], [33], [36]	278	282	0.64	0.46 to 0.91	0.01	0%
2 yr OS	6 [6], [17], [18], [31]–[33]	233	236	0.36	0.22 to 0.59	<0.0001	0%
3 yrOS	4 [17], [20], [31], [37]	154	136	0.37	0.20 to 0.70	0.002	13%
ORR	11 [6], [18]–[20], [31]–[36], [38]	401	410	0.58	0.44 to 0.78	0.0003	0%
DCR	10 [6], [18]–[20], [31]–[34], [36], [38]	369	378	0.41	0.29 to 0.58	<0.00001	0%

Forest plot comparing the 1-, 2- and 3-year OS between the non-CIK and CIK groups.OR, odds ratio; OS, overall survival. Due to the low heterogeneity detected, the fixed-effect model was used in this OS meta-analysis. Comparison of the ORR and the DCR between the non-CIK group and CIK group. OR, odds ratio; ORR, objective response rate; DCR, disease control rate. Due to the lack of heterogeneity, the fixed-effect model was used. OS: overall survival; ORR: objective response rate; DCR: disease control rate.

Concerning the median PFS, the CIK group did not produce any significant improvement compared with the corresponding control groups (95%CI −13.27 to 3.89, *p* = 0.28), whereas the median TTP clearly prolonged the median time to disease progression in the CIK group (95%CI −2.70 to −0.47, *p* = 0.005) (Table 3).

### Response rate

The CIK cell-based therapy group showed favorable results when subjected to both analysis of the ORR (OR 0.58, 95%CI 0.44–0.78, *p* = 0.00003) and the DCR (OR 0.41, 95%CI 0.29–0.58, *p*<0.0001), compared with the corresponding control arms. With no significant heterogeneity, a fixed-effect model was used in the ORR and DCR analyses (Table 4). Cochran's Q test resulted in a statistically significant P-value, and the corresponding quantity for I^2^ was 0% for both groups, indicating that there was no evidence of heterogeneity among the individual studies.

### Immunological assessment of T-cell subgroups

When heterogeneity was observed in the T-cell subgroups, a random-effects model was applied for the overall and subgroup analysis of T-cell immunological assessments (Table 5). The results demonstrated a substantially increased ratio of CD3^+^ (MD 8.21, 95%CI 5.79–10.64, *p*<0.00001), CD4^+^ (MD 5.59, 95%CI 4.10–7.07, *p*<0.00001), CD4^+^CD8^+^ (MD 0.49, 95%CI 0.37–0.61, *p*<0.00001), CD3^+^CD56^+^ (MD 7.80, 95%CI 2.61–12.98, *p* = 0.003) and NK cells (CD3^−^CD16^+^CD56^+^) (MD 6.21, 95%CI 2.25–10.17, *p* = 0.002), whereas the ratio of CD3^+^CD8^+^ (MD 2.55, 95%CI −2.46 to 7.56, *p* = 0.32) generated no statistical improvement after CIK treatment. In addition, the pooled analysis showed a significant decrease in the percentage of CD8^+^ (MD −2.75, 95%CI −3.88 to −1.63, *p*<0.00001) and Treg (CD4^+^CD25^+^CD127^−^) (MD −1.26, 95%CI −1.94 to −0.58, *p* = 0.0003) subgroups after treatment with CIK cell-based therapy.

**Table 5:** Comparison of CD3^+^, CD4^+^, CD3^+^CD8^+^, CD4^+^CD8^+^, CD3^+^CD56^+^, NK, CD8^+^ and Treg before CIK treatment and after CIK therapy.

Event	No. of Trials [Ref]	No. of pts Before-CIK CIK		Mean Difference	95% CI	P value	Heterogeneity (I^2^)
CD3^+^	9 [6], [17], [20], [21], [31]–[33], [35], [36]	359	359	8.21	5.79 to 10.64	<0.00001	67%
CD4^+^	5 [6], [21], [31], [32], [35]	174	174	5.59	4.10 to 7.07	<0.0001	0%
CD3^+^CD8^+^	4 [17], [18], [33], [36]	174	174	2.55	−2.46 to 7.56	0.32	89%
CD4^+^CD8^+^	4 [6], [31], [32], [35]	144	144	0.49	0.37 to 0.61	<0.00001	53%
CD3^+^CD56^+^	6 [6], [18], [19], [30], [36], [38]	222	222	7.80	2.61 to 12.98	0.003	99%
NK	4 [6], [21], [32], [36]	154	154	6.21	2.25 to 10.17	0.002	90%
CD8^+^	5 [6], [21], [31], [32], [35]	174	174	−2.75	−3.88 to −1.63	<0.00001	0%
Treg	3 [17], [33], [36]	153	153	−1.26	−1.94 to −0.58	0.0003	58%

Forest plot for the comparison of T-cell subgroups, before and after treatment with the CIK cell-based therapy. The random-effects meta-analysis model was used in this analysis.

### Immunological assessment of Ag-NORs and CEA expression

Due to the limited data presented in the published papers, only some of the immunological assessments, e.g., Ag-NORs (argyrophilic nucleolar organizer regions), and NSCLC tumor markers, e.g., CEA, were subjected to analysis. Heterogeneity was observed, and a random-effects model was therefore applied for the analysis of the subgroups and the overall analysis. The analysis showed that the CIK group significantly improved the patients' T lymphocyte immune activity, showing better Ag-NORs (MD −0.71, 95%CI −0.94 to −0.47, *p* = 0.00001) compared with the non-CIK therapy group (Table 6). The CEA expression level in the analysis was based on two trials [38], [39]. The plasma CEA was markedly decreased in the CIK group compared to the non-CIK group (MD 3.96, 95%CI 1.64–6.28, *p* = 0.0008) (Table 6).

**Table 6:** Comparison of the immunological assessment of Ag-NORs and CEA expression between the CIK and non-CIK group.

Event	No. of Trials [Ref]	No. of pts Non-CIK CIK		Mean Difference	95% CI	P value	Heterogeneity (I^2^)
Ag-NORs	2 [20], [38]	69	68	−0.71	−0.94 to −0.47	0.00001	33%
CEA	2 [38], [39]	47	47	3.96	1.64–6.28	0.0008	0%

Summary of the significant points in the Ag-NORs and CEA expression level between the CIK group and the non-CIK group with meta-analysis. The random-effects model was used for the calculations. Ag-NORs: argyrophilic nucleolar organizer regions; CEA: carcinoembryonic antigen; Pts: patients; 95%CI: 95% confidence interval; significant difference: P value <0.05.

### Toxicity and adverse reactions

**Figure 3:**
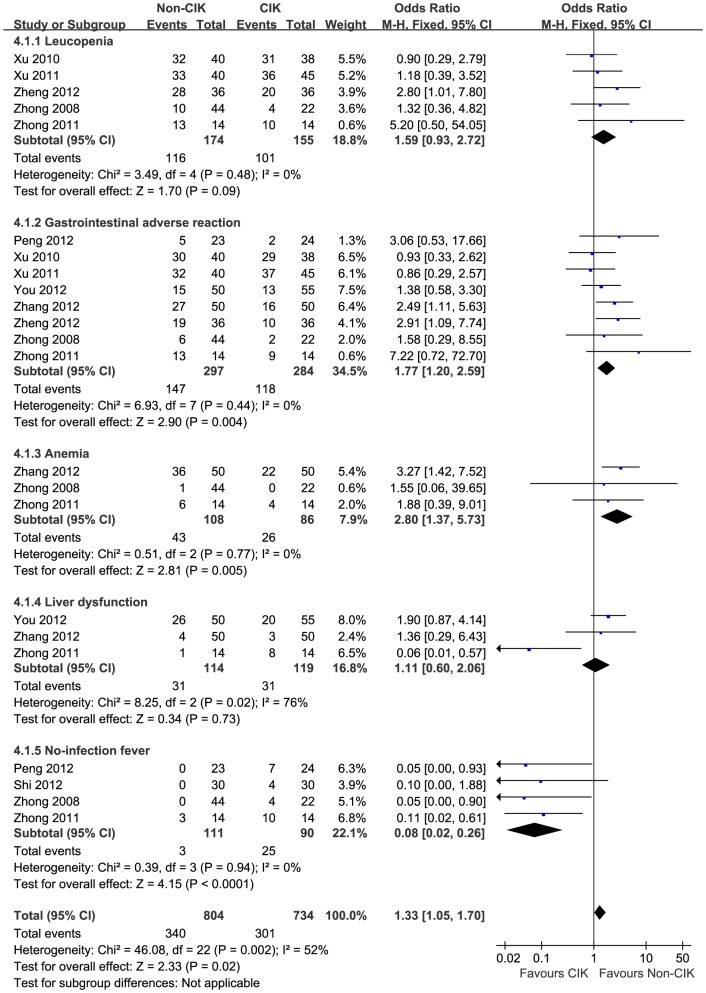
Forest plot comparing the toxicity and no treatment-related side effects between the CIK group and the non-CIK group. Some serious adverse effects were observed significantly less frequently in the CIK group. Due to the lack of heterogeneity, the fixed-effect model was used.

The patients in the CIK group observed fewer severe side effects from chemotherapy, such as fewer cases of grade III and IV leucopenia, gastrointestinal adverse reactions, anemia and liver dysfunction (Figure 3). Without significant heterogeneity, a fixed-effect model (Mantel-Haenszel method) was used for the side effect analysis.

After CIK cell transfusion, most of the patients developed a slight fever, between 37.5 and 39 degrees, but the patients recovered within a few days without severe side effects. Four types of serious chemotherapy side effects could lead to toxic reactions in both groups of patients. The pooled analysis showed that the adverse effects of gastrointestinal adverse reactions (OR 1.77, 95%CI 1.20–2.59, *p* = 0.004) and anemia (OR 2.80, 95%CI 1.37–5.73, *p* = 0.005) generated a significant difference, with fewer episodes in the CIK group. Leucopenia and liver dysfunction were observed less frequently in the patients receiving the CIK treatment, but neither set of data displayed a significant difference compared with the non-CIK group (OR 1.59, 95% CI 0.93–2.72, *p* = 0.09; OR 1.11, 95%CI 0.60–2.06, *p* = 0.73).

## Discussion

Immunotherapy has benefited from an increased understanding of tumor immunology and genetics. A number of studies have confirmed that immunotherapy is a safe and feasible treatment option for cancer patients [12]–[16]. Therefore, conventional therapy combined with adoptive cell immunotherapy is associated with a favorable prognosis compared to chemotherapy alone [18]. Our analysis was designed to elucidate the effects of CIK cell therapy on improving the therapeutic efficacy and safe treatment of NSCLC patients based on a variety of evaluation indexes, including clinical survival outcomes, clinical response rates, immunophenotypes and adverse effects.

In our study, 17 trials were selected for the analysis of the culture of CIK cells and treatment regimens. Most of the trials collected 50–100 ml of autologous peripheral blood and separated the mononuclear cells for further induction. Some of the necessary cytokines were supplied to the cultures of CIK cells, such as IL-2, IFN-γ, and CD3mAb, in 1640 or serum-free medium. Based on our study, most of the treatments with repetitive infusions of 1×10^9^ to 2×10^12^ CIK cells were administered for at least 2 weeks on every second day for a minimum of two treatment cycles. However, the different doses and cycles of CIK cell transfusions may lead to different outcomes and immune responses.

In the present study, the CIK cell-based therapy group was associated with favorable results based on an evaluation of both the overall survival and clinical responses (Table 4). The 1-year survival (OR 0.64, 95%CI 0.46–0.91, *p* = 0.01), 2-year survival (OR 0.36, 95%CI 0.22–0.59, *p*<0.0001), and 3-year survival (OR 0.37, 95%CI 0.20–0.70, *p* = 0.002) showed significantly prolonged durations in the CIK cell therapy group. A favorable DCR and ORR were also observed in patients receiving CIK cell therapy (p<0.0001). The MTTP and MST also showed significant improvements in the CIK group (*p* = 0.005, *p* = 0.02). CIK cells, which are also known as NKT cells, exhibit both the cytotoxicity activities of T-lymphocytes and the restrictive tumor-killing activity by non-MHC of NK cells, among which the main effectors are CD3^+^CD56^+^ cells [7]. In total, 4 of 17 trials used DCs pulsed with lung cancer antigens or tumor lysate, whereas 9 trials used mature DCs co-cultured with CIK cells (Table 2). DCs possess antigen-presenting activities on the extracellular surface and are able to activate the proliferation of T cells and CIK cells. Therefore, considering the poor immunogenicity of NSCLC, CIK infusion with an immunoadjuvant or tumor-specific antigen pulsed DCs boosted the immune responses [24]. Therefore, CIK cell-based therapy even acting through completely different mechanisms for fighting cancer cells, can lead to an improvement in the clinical objective responses based on the assessment of traditional RECIST criteria [40].

The human immune response against cancer cells is mainly dependent on cellular immunity. Previous studies have found that the numerical ratios of T-lymphocyte subsets in the peripheral blood are disordered in tumor patients [17]. In the present study, we observed a substantially increased percentage of CD3^+^ and CD4^+^ (*p*<0.001), the ratio of CD4^+^CD8^+^ and CD3^+^CD56^+^ (*p*<0.001) and NK cells (*p* = 0.002), but a significant decrease in the percentage of the CD8^+^ (*p*<0.001) and Treg (*p* = 0.0003) subgroups after DC-CIK treatment by meta-analysis. Many studies have demonstrated that CIK cells possess strong cytotoxicity against a variety of T-lymphocyte populations, among which CD3^+^CD56^+^ is mainly responsible for the MHC unrestricted antitumor activity [8]. In addition, the number of CD4^+^ and CD8^+^ T-cells plays an important role in affecting clinical outcomes in NSCLC. The activation of CD4^+^ T cells contributes to the secretion of immune regulatory cytokines, including IL-2, IL-12, and IFN-γ, which in turn facilitate an elevation in the cytolytic CD8^+^ T cell responses, thereby inducing tumor cell death [25]. The activation of CD4^+^ T cells also enhances the killing activity of NK cells and the phagocytic activity of macrophages, triggering a humoral immune response that leads to antibody production, thus CD4^+^ and CD8^+^ have a synergistic relationship in immune responses. Our meta-analysis demonstrated that CD3^+^, CD4^+^, CD4^+^CD8^+^, CD3^+^CD56^+^ and NK cells were increased after DC-CIK treatment, therefore suggesting the improvement of immune function after immunotherapy in the NSCLC patients.

In addition, we should note that CD8^+^ T cells were not significantly increased after the immunotherapy, which also showed the varied immunophenotypes compared with the results of other T-cell assessments by the CIK treatment in different solid carcinomas [11]–[13]. Naïve CD4^+^ T lymphocytes undergo cell differentiation in the presence of antigen, co-stimulatory molecules and cytokines, and these cells can be divided into several major groups: Th1, Th2 and Treg cells [26]. Th1 helper cells are the host immunity effectors against intracellular bacteria and protozoa. These are triggered by IL-12, IL-2 and the effector cytokine IFN-γ. The main effector cells of Th1 immunity are macrophages, CD8 T cells, IgG B cells, and CD4 T cells. Th2 helper cells are the host immunity effectors against multicellular helminthes [26]. The main effector cells are eosinophils, basophils, and mast cells, as well as IgE B cells and IL-4/IL-5 CD4 T cells [27]. T regulatory cells express FoxP3 and produce TGF-β and CD4^+^CD25^+^CD127^−^ T subgroups to suppress immune responses against Th1 and Th2. In addition, tumor cells also express high levels of CD4^+^CD25^+^ Treg cells, which help direct immunosuppressive cytokines to the tumor microenvironment [28], so the decrease of the Treg cell may be helpful to remove the immunosuppressive effect for NSCLC patients, and our results also demonstrated a lower number of Treg cells. Higher proportions of Treg and proliferating CD8^+^ T cells were both associated with poor survival in malignancies lung cancer [41], suggesting that DC-CIK immunotherapy may play a role in enhancing the immune function of NSCLC patients.

Immunotherapy exerts its effect on the cellular immune response and requires time for immune cytokines to change the tumor burden or survival time. In our present study, we also evaluated T lymphocyte immune activity by Ag-NORs *in vivo* and the NSCLC tumor marker CEA. The significant increase in Ag-NORs (*p* = 0.00001) and the reduction in the CEA content (*p* = 0.0008) observed in the CIK group contributed to the prevention of short-term recurrence and improvement of clinical responses. We also analyzed clinical survival outcomes, clinical response rates, immunophenotypes and tumor markers, and we hypothesized that the CIK cells fight with tumor cells in several different ways, including direct cellular interactions (Fas/FasL pathway, granzyme B), the secretion of cytokines (IFN-γ, TNF-α, IL-2) and antibodies, and immune response regulations (T-lymphocyte variations) [29]. In all, our meta-analysis evaluated a variety of T-cell subgroups, and the differences in the cytokines used for immunotherapy, and we found that the results were consistent with the clinical therapeutic outcomes, such as the overall survival and clinical response.

In our analysis, CIK cell-based therapy yielded a disappointing result in non-infective fever (P<0.0001), and no other major side effect was observed. The pooled analysis showed that the adverse effects of gastrointestinal adverse reactions (*p* = 0.004) and anemia (*p* = 0.005) generated significant differences with fewer episodes in the CIK group. Thus, CIK cell immunotherapy with chemotherapy has proven to be a feasible and effective method for the treatment of NSCLC without severe side effects.

### Limitation of the study

The 17 trials included in this meta-analysis were selected with an RCT to improve statistical reliability. To avoid bias in the identification and selection of trials, we minimized the possibility of overlooking published papers to the greatest extent. Although we selected using RCT as much as possible, there are some major criteria that did not receive a good grade under the Jadad scale, such as allocation concealment and intention-to-treat, meaning our study may have a moderate risk of bias. We also used a funnel plot to evaluate the publication bias. In our analysis, overall survival, clinical response rate, and side effects suffered low published bias; however, immunological assessment and T cell subgroups observed a high published bias. Therefore, there are some limitations to our study. First, CIK cell-based therapy is a greater concern for Chinese scholars; therefore, all 17 selected trials were from Asia, because there is a global lack of any multinational large-sample multicenter clinic research regarding CIK cell therapy for NSCLC. Second, some of the papers had to be excluded due to the lack of a control arm during the experimental design; however, some of the papers produced even better prognosis after the CIK treatment. Third, our analyzed data were selected from published papers rather than drawn first-hand from patient records, potentially causing an overestimation of the analytical results. Therefore, only the enrollment of a larger sample could minimize this bias. However, various crucial issues for CIK cell-based immunotherapy need to be conquered before it can be approved as a standard treatment for NSCLC tumors due to several obstacles. First, the different dosage and treatment regimens of CIK cell transfusions may lead to different outcomes and immune responses. Second, although most of our selected papers focused on therapeutic outcomes based on chemotherapy RECIST criteria, due to the different tumor killing mechanisms, a novel immune-related response criterion (irRC) should also be used for the assessment of immunotherapy clinical activities [40]. Third, due to the poor immunogenicity of NSCLC, optimizing DC modifications combined with CIK cell infusion may contribute to more favorable clinical outcomes in NSCLC patients.

Taken together, the CIK-combined therapy for NSCLC presented a significantly prolonged overall survival, an improved clinical response rate, a strengthened immune system, and low rates of adverse side effects. The CIK therapy is more concerned with reducing the tumor burden stage than curing cancer. The CIK adoptive immune therapy showed potential regarding improved clinical outcomes, and there is increasing evidence that the CIK therapy treatment of NSCLC evokes specific humoral and cellular antitumor immune responses. However, the timing of the immunotherapy, dosage, regimens and efficient tumor antigens still require further research.

## Conclusion

In total, 17 randomized controlled trials of NSCLC with 1172 patients were included in the present analysis. Combined CIK cell therapy for the treatment of NSCLC demonstrated significant superiority in terms of overall survival and objective response compared with the non-CIK group. The T-lymphocyte subgroups also seemed to favorably affect the immune system after chemotherapy. The data also indicated that CIK therapy relieves the side effects of chemotherapy without causing any additional major side effects aside from non-infective fever. This analysis supports a further larger-scale meta-analysis for the evaluation of the efficacy of CIK adoptive cell therapy for the treatment of NSCLC in the future.

## Supporting Information

Checklist S1 **PRISMA Checklist.**
(DOCX)

File S1 **Language edit certification (Figures S1A.tif and Figures S1B.tif).**
(TIF)

File S2 **References specification.**
(PDF)
