# Supplementary material for: Adoptive Immunotherapy of Cytokine-Induced Killer Cell Therapy in the Treatment of Non-Small Cell Lung Cancer
Source: PLoS One. 2014 Nov 20;9(11):e112662. doi: 10.1371/journal.pone.0112662 (PMC4239020; doi:10.1371/journal.pone.0112662)
Supplement: Checklist S1 — PRISMA Checklist. (DOCX) [file pone.0112662.s001.docx]

| **Section/topic** | **#** | **Checklist item** | **Reported on page #** |
| --- | --- | --- | --- |
| **TITLE** | | | 1 |
| Title | 1 | Adoptive immunotherapy of cytokine-induced killer cell therapy in the treatment of non-small cell lung cancer in China (this is a systematic review and meta-analysis) | 1 |
| **ABSTRACT** | | | 2 |
| Structured summary | 2 | **Aim.** The aim of this study was to systemically evaluate the therapeutic efficacy of cytokine-induced killer (CIK) cells in the treatment of non-small cell lung cancer.  **Materials and methods.** A computerized search of randomized controlled trials for CIK cell-based therapies was performed. Overall survival, clinical response rate, immunologic assessment and side effects were evaluated.  **Results.** Overall, 17 randomized controlled trials of non-small cell lung cancer (NSCLC) with a total of 1172 patients were included in the present analysis. Our study showed that the CIK group significantly improved the objective response rate and overall survival compared to the Non-CIK group. We observed substantially increased percentages of CD3+, CD4+, CD4+CD8+, CD3+CD56+ and NK cells, whereas significant decreases were noted in the percentage of CD8+ and regulatory T cell (Treg) subgroups after CIK combined therapy. A significant increase in the Ag-NORs was observed in the CIK-treated patient group (p=0.00001), whereas CEA was more likely reduced to a normal level after CIK treatments (p=0.0008). Without major side effects, only the incidence of fever in the CIK group was significantly higher compared to chemotherapy alone.  **Conclusion.** The CIK combined therapy demonstrated significant superiority in overall survival, clinical response rate, and T lymphocytes and lacked any evidence of major adverse events in patients with NSCLC. | 2 |
| **INTRODUCTION** | | | 3-4 |
| Rationale | 3 | A growing number of clinical trials have suggested that CIK therapy yields highly compelling objective clinical responses in several solid carcinomas. These studies showed that the immunotherapy of cancers with CIK cells may improve immunologic and clinical responses, promote the quality of life (QoL) of cancer patients, and extend their life spans under certain conditions. However, there is no systematic review to assess the therapeutic efficacy of CIK cell therapies combined with chemotherapy in NSCLC. | 3-4 |
| Objectives | 4 | We performed a systematic meta-analysis on CIK therapy with randomized controlled trials on NSCLC. Our large-scale CIK immunotherapy clinical trials systematically analyzed the clinical efficiency and safety, considering overall survival, clinical response, immunologic assessments and side effects. | 4 |
| **METHODS** | | | 5-7 |
| Protocol and registration | 5 | The analysis was performed using a Review Manager Version 5.0 (Nordic Cochran Centre, Copenhagen). To assess statistical heterogeneity between the studies, the Cochran’s chi-square (Q-test) was performed, with a predefined significance threshold of 0.1. | 5 |
| Eligibility criteria | 6 | The selection criteria were as follows: (1) English language studies on human clinical trials with patients at all stages of NSCLC were included; (2) RCT with CIK-based immunotherapy combined with thermotherapy versus chemotherapy alone for the treatment of NSCLC alone were included; (3) all trials approved by the local ethical committee with all patients signing a study-specific consent form prior to study entry were included; (4) case studies, review articles, and studies involving fewer than 10 patients were excluded; (5)uncontrolled metabolic disease, inadequate hepatic function, renal dysfunction, neurological disorders and other infectious diseases were excluded in the study; and (6) blood samples receiving any chemotherapy or radiotherapy in one month before the treatment were excluded. | 6 |
| Information sources | 7 | Relevant studies were identified by searching PubMed, the Cochrane Center Register of Controlled Trials, ScienceDirect, Embase, and China National Knowledge Infrastructure for randomized controlled trials (RCT) in the most recent decades. In addition, we manually searched a website of clinical trials for ongoing trials. Reference lists of previously published trials and relevant review articles were examined for other eligible trials. we performed manual searches in reference lists and conference proceedings of the American Society of Clinical Oncology (ASCO) Annual Meetings and the European Cancer Conference (ECCO) | 5 |
| Search | 8 | Keywords used included ’non-small-cell lung cancer’, ‘adoptive immunotherapy’, and ‘cytokine induced killer cells’ Adoptive immunotherapy arms with no adjuvant treatment in NSCLC patients except undergone the same chemotherapy compared with control arms. | 5 |
| Study selection | 9 | No language restriction was applied. We excluded abstracts that were never subsequently published as full papers and studies on animals and cell lines.  Data searches yielded 167 references, 91 of which were considered ineligible for different reasons (44 non-CIK immunotherapy, 19 multiple cancer analysis, 18 reviews, and 10 animal models). The rest of the 76 articles were further evaluated; 59 trials were excluded due to language, lack of an RCT, and insufficient data reasons. The final 17 articles were included in the meta-analysis with RCTs of CIK cell-based therapies for the treatment of NSCLC. The quality assessment of the 17studies is summarized in Table1. | 5 and 8 |
| Data collection process | 10 | We extracted the data mannualy, contacted author, asked experts in the field, and performed manual searches in reference lists. Data extraction was independently conducted by two reviewers (Min Wang and Jun-Xia Cao) using a standardized approach. Disagreement was adjudicated by a third reviewer (Zheng-Xu Wang) after referring back to the original publications. | 5-6 |
| Data items | 11 | The primary clinical endpoints in RCT for cancer therapies employed the measure of median survival time (MST) and progression-free survival (PFS), time to progression (TTP) and overall survival (OS). The secondary endpoints were the clinical response rate, including the objective response rate (ORR) and disease control rate (DCR). ORR was defined as the sum of partial rates (PRs) and complete response rates (CRs), and DCR, as the sum of stable disease (SD), PR and CR, according to the World Health Organization criteria. Side effects and toxicity were graded according to the National Cancer Institute Common Toxicity Criteria. | 6-7 |
| Risk of bias in individual studies | 12 | To ascertain the validity of eligible randomized trials, pairs of reviewers working independently. The overall quality of each involved paper was evaluated by the Jadad Scale. A few of the major criteria were employed as a grading scheme: (1) randomization; (2) allocation concealment; (3) blinding; (4) lost to follow up; (5) ITT (intention to treat); and (6) Baseline. We also used a funnel plot to evaluate the publication bias. | 6 |
| Summary measures | 13 | The fixed (or random)-effects meta-analysis model (Mantel-Haenszel method) was used.The odds ratios (OR) were the principal measurements of effect and were presented with a 95% confidence interval (CI). P-values of 0.05 were considered to be statistically significant. All reported P-values resulted from two-sided version tests of the respective tests. | 7 |
| Synthesis of results | 14 | To assess statistical heterogeneity between the studies, the Cochran’s chi-square (Q-test) was performed, with a predefined significance threshold of 0.1. | 7 |

Page 1 of 2

| **Section/topic** | **#** | **Checklist item** | **Reported on page #** |
| --- | --- | --- | --- |
| Risk of bias across studies | 15 | The symmetry of such ‘funnel plots’ was assessed both visually to see if the effect decreased with increasing sample size. | 6 |
| Additional analyses | 16 | The Cochran’s chi-square (Q-test) was performed and the treatment effects were examined according to quality components. | 7 |
| **RESULTS** | | | 8-12 |
| Study selection | 17 | Data searches yielded 167 references, 91 of which were considered ineligible for different reasons (44 non-CIK immunotherapy, 19 multiple cancer analysis, 18 reviews, and 10 animal models). The rest of the 76 articles were further evaluated; 59 trials were excluded due to language, lack of an RCT, and insufficient data reasons. The final 17 articles were included in the meta-analysis with RCTs of CIK cell-based therapies for the treatment of NSCLC. See Figure 1 | 7 |
| Study characteristics | 18 | The characteristics of the 17 trials are listed in Table 2. Our selected 17 trials with a number of 1172 NSCLC patients in stage I-IV were included in the present analysis, and 90% of them included metastatic or locally advanced NSCLC. The enrolled ages were between 28 and 82 years of age, with a median age greater than 50.  In all 17 trials, the control arm was chemotherapy or cyberknife alone, whereas the treatment arm was chemotherapy or cyberknife combined with CIK cell therapy. In each trial, all patients in the CIK group were treated identically to those in the chemotherapy group in terms of chemotherapy doses and cycles. In all 17 trials of the treatment arm, most of the trials were treated with CIK cells plus DC immunotherapy combined with chemotherapy, although the patients in four of the trials were injected with CIK cells combined with chemotherapy [6,30,38,39]. Most of the CIK groups used DCs without impulse, i.e., the DCs were only induced to mature before co-culture with CIK cells. In 4 out of 17 studies, the DCs were injected while being pulsed with lung cancer antigens or tumor lysate [17,22,33,37]. Some of the necessary cytokines were supplied in a culture of CIK, IL-2, IFN-γ, and CD3mAb in a variety of culture media. The patients received cell infusions of 1X109 to 2X1012 cells per course, mostly at a 109order of magnitude. Most of the treatments with repeated CIK cell infusions were administered for at least 2 weeks, and some of them lasted over1month. The injected route for immunotherapy was mainly intravenous for CIK cells and via subcutaneous for DCs. | 8-9 |
| Risk of bias within studies | 19 | The overall quality of each involved paper was evaluated by the Jadad Scale. The quality assessment of the 17 studies is summarized in Table1. We also used a funnel plot to evaluate the publication bias. In our analysis, overall survival, clinical response rate, and side effects suffered low published bias. However, immunological assessment and T cell subgroups observeda high published bias (graph not shown). | 8 |
| Results of individual studies | 20 | See Figure 2, 3, 4, and 5. | 9-12 |
| Synthesis of results | 21 | Our study showed that the CIK group significantly improved the objective response rate and overall survival compared to the non-CIK group. We observed substantially increased percentages of CD3+, CD4+, CD4+CD8+, CD3+CD56+ and NK cells, whereas significant decreases were noted in the percentage of CD8+ and regulatory T cell (Treg) subgroups after CIK combined therapy. A significant increase in the Ag-NORs was observed in the CIK-treated patient group (p=0.00001), whereas CEA was more likely reduced to a normal level after CIK treatments (p=0.0008). Without major side effects, only the incidence of fever in the CIK group was significantly higher compared to chemotherapy alone. | 9-12 |
| Risk of bias across studies | 22 | Strong evidence of heterogeneity (I2 >50%, P<0.05) was observed. To explore this heterogeneity, a funnel plot was drawn. The funnel plot shows evidence of considerable asymmetry. | 9-12 |
| Additional analysis | 23 | Give results of additional analyses, if done (e.g., sensitivity or subgroup analyses, meta-regression [see Item 16]). | Not available |
| **DISCUSSION** | | | 12-17 |
| Summary of evidence | 24 | Overall, our study suggests that DC-CIK cells have a smooth superiority in prolonging the survival time, enhanced the immunity function and improved the efficacy of the treatment in NSCLC patients. | 12-16 |
| Limitations | 25 | Although we selected using RCT as much as possible, there are some major criteria that did not receive a good grade under the Jadad Scale, such as allocation concealment and intention-to-treat, which means that our study may have a moderate risk of bias. We also used a funnel plot to evaluate the publication bias. In our analysis, overall survival, clinical response rate, and side effects suffered low published bias; however, immunological assessment and T cell subgroups observeda high published bias (graph not shown).  Therefore, there are some limitations to our study. First, CIK cell-based therapy is a greater concern for Chinese scholars; therefore, all 17 selected trials were from Asia, because there is a global lack of any multinational large-sample multicenter clinic research regarding CIK cell therapy for NSCLC. Second, some of the papers had to be excluded due to the lack of a control arm during the experimental design; however, some of the papers produced even better prognosis after the CIK treatment. Third, our analyzed data were selected from published papers rather than drawn first-hand from patient records, potentially causing an overestimation of the analytical results. Therefore, only the enrollment of a larger sample could minimize this bias. | 16-17 |
| Conclusions | 26 | In total, 17 randomized controlled trials of NSCLC with 1172 patients were included in the present analysis. CIK combined therapy in NSCLC demonstrated significant superiority in overall survival and objective response compared with the non-CIK group. T-lymphocyte subgroups also seemed to favorably affect the immune system after chemotherapy. The data also indicated that the CIK therapy relieved the side effects of chemotherapy without causing any additional major side effects, aside from non-infective fever. This analysis supports a further larger scale meta-analysis to evaluate the efficacy of CIK adoptive cell therapy in NSCLC in the future. | 17 |
| **FUNDING** | | | 18 |
| Funding | 27 | This research work was supported by the National Natural Science Foundation of China (No. 31171427 and 30971651 to Zheng-Xu Wang, and No. 30700974 to Jun-Xia Cao), Beijing Municipal Science & Technology Project; Clinical characteristics and Application Research of Capital (No. Z121107001012136 to Zheng-Xu Wang) and the Postdoctoral Foundation of China (No. 20060400775 to Jun-Xia Cao). | 18 |

*From:*  Moher D, Liberati A, Tetzlaff J, Altman DG, The PRISMA Group (2009). Preferred Reporting Items for Systematic Reviews and Meta-Analyses: The PRISMA Statement. PLoS Med 6(6): e1000097. doi:10.1371/journal.pmed1000097

For more information, visit: **www.prisma-statement.org**.

Page 2 of 2
