# Supplementary figures and images for: Adoptive Immunotherapy of Cytokine-Induced Killer Cell Therapy in the Treatment of Non-Small Cell Lung Cancer
Source: PLoS One. 2014 Nov 20;9(11):e112662. doi: 10.1371/journal.pone.0112662 (PMC4239020; doi:10.1371/journal.pone.0112662)

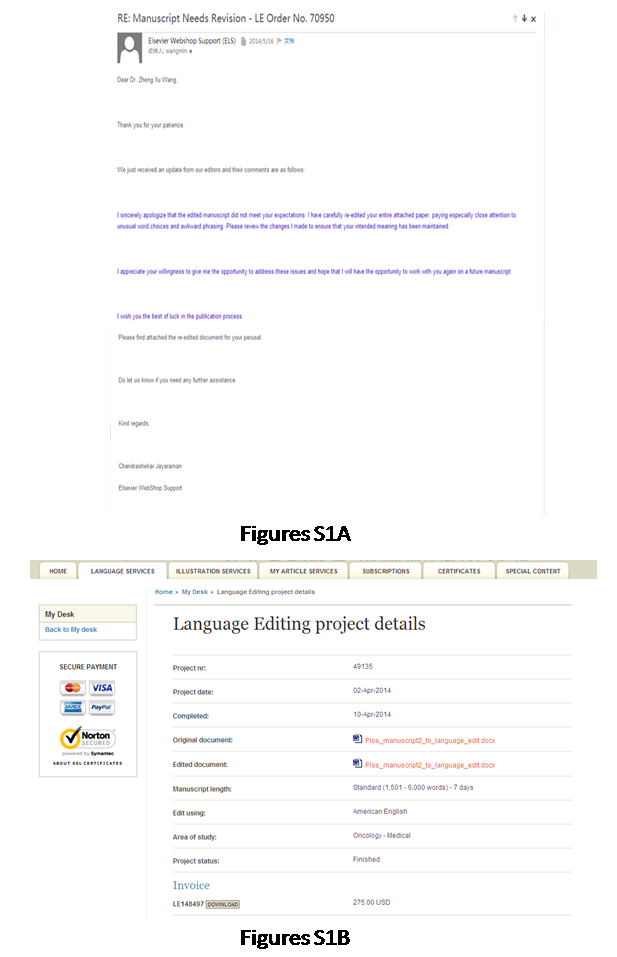

Supplement: File S1 — Language edit certification (Figures S1A.tif and Figures S1B.tif). (TIF) [file pone.0112662.s002.tif]
